# Association between Neurocognitive Impairment and the Short Allele of the 5-HTT Promoter Polymorphism in Depression: A Pilot Study

**DOI:** 10.1155/2013/849346

**Published:** 2012-12-18

**Authors:** Hely Kalska, Ullamari Pesonen, Sanna Lehikoinen, Jan-Henry Stenberg, Jari Lipsanen, Jussi Niemi-Pynttäri, Arja Tuunainen

**Affiliations:** ^1^Institute of Behavioural Sciences, University of Helsinki, P.O. Box 9, 00014 University of Helsinki, Finland; ^2^Department of Pharmacology, Drug Development and Therapeutics, University of Turku, 20014 Turku, Finland; ^3^Department of Psychiatry, Helsinki University Central Hospital, P.O. Box 590, 00029 HUS, Finland; ^4^Department of Psychiatry, University of Helsinki, P.O. Box 22, 00014 University of Helsinki, Finland

## Abstract

Depression has been shown to be associated with cognitive deficits in various cognitive domains. However, it is still unclear which factors contribute to cognitive impairment. The objective of this study was to find out whether a functional polymorphism in the promoter region of the serotonin transporter (5-HTTLPR) gene is associated with the impairment of cognitive functioning among depressed patients. In a pilot study, a sample of 19 patients with major depressive disorder (MDD) and 19 healthy controls was investigated with an extensive psychiatric and neuropsychological examination. All participants were genotyped for 5-HTTLPR. Depressed patients with the short allele of the 5-HTT promoter region exhibited inferior cognitive performance compared to patients with the long allele polymorphism. In healthy controls, no association between genotype and cognitive performance was found. The result suggests that in MDD patients with the short allele of the 5-HTTLPR polymorphism the vulnerability to cognitive impairment is increased compared to MDD patients without the short allele inheritance. These preliminary findings need to be confirmed in a larger cohort of MDD patients.

## 1. Introduction

It is well established that patients with depression are subject to multiple cognitive deficits. The impairments have been found in a broad range of neuropsychological tests and in various cognitive domains, such as executive functions, psychomotor speed, and episodic memory [1]. Impairment may occur in patients under medication as well as in drug-free patients [2], in younger or elderly patients [3, 4] or in different depression severity levels or subtypes. Some of these deficits may persist even upon clinical recovery [5]. Most importantly, not all of the depressed patients show deterioration in cognitive capabilities, that is, some patients seem to be more vulnerable to impairment than others. The complex relationship between neurocognitive function and mood may partly be the result of interaction between the serotonergic system and the corticolimbic neural circuits of these processes [6]. Growing literature indicates the importance of the serotonin transporter gene (*SLC6A4*), which codes for the serotonin transporter protein (5-HTT), in the development and integrity of neural systems that subserve emotional regulation [7]. Recent research has provided important insights into the role of genetic variation in the *SLC6A4*-linked polymorphic region (5-HTTLPR) on neural systems subserving anxiety and depression. The short variant of the 5-HTTLPR of *SLC6A4 *(S) has been associated with traits related to anxiety and depression [8]. The short variant S has also been reported being associated with susceptibility to depression in response to stressful life events [9] and in response to increased stress sensitivity in the childhood maltreatment [10]. It has been suggested that the 5-HTT gene might also be involved in social behavior [11]. Whether 5-HTTLPR exacerbates cognitive impairment in association with depression is still unknown. It has been shown that the 5-HTTLPR polymorphism has an effect on hippocampal volumes of depressed patients, which is apparent only in *S/S* genotype [12]. It has also been implied that major depressive disorder (MDD) could serve as a risk factor for developing Alzheimer's disease [13]. On the other hand, there is some evidence of better cognitive performance among healthy individuals possessing a copy of the short variant of the polymorphism compared with individuals homozygous for the long variant [14, 15]. The evidence concerning the role of 5-HTT in depression-related cognitive functioning is sparse. In our naturalistic sample of patients with MDD, the aim was to find out whether variation in the serotonin transporter gene moderates the influence of depression on cognitive impairment.

## 2. Methods

### 2.1. Participants

Nineteen inpatients, who met DSM-IV criteria for major depressive disorder (MDD), were recruited from the Department of Psychiatry of the Helsinki University Central Hospital. We included patients with a current moderate or severe episode of MDD with a minimum score of 18 points on the Hamilton Rating Scale for Depression (HDRS, 21 items) [16]. Diagnoses were made using the Structured Clinical Interview for DSM-IV Axes I and II Disorders (SCID-I and SCID-II) [17, 18]. Exclusion criteria were current or past neurological disorders (except for occasional migraine attacks), drug- and/or alcohol-dependence disorders within the last five years, and overt psychosis during the study. All patients were on antidepressant medication; of these, only eight patients with one antidepressant only. Although the patients were primarily on selective serotonin reuptake inhibitor (such as fluoxetine) or on serotonin and norepinephrine reuptake inhibitor (such as venlafaxine) medication, sedative antidepressant medication (such as mirtazapine or mianserin) was also used. Prescriptions of additional psychiatric drugs (such as antipsychotics for mood or sleep and mood stabilizer for pain) were in regular use for some of the patients. Six patients were habitual smokers, smoking daily or almost daily. Nineteen healthy volunteers, serving as control subjects and recruited by flyers from various locations, such as educational communities, schools, and business companies, were free from current or past neurological, mental, and alcohol-dependence disorders and were not on psychotropic medication, neither did their first-degree relatives have a history of mental illness. All participants were Caucasian. The study was approved by the Ethics Committee of the Department of Psychiatry, Helsinki University Central Hospital. Written informed consent was obtained from all participants.

### 2.2. Genotyping

5-HTTLPR (SLC6A4, 44-BP INS/DEL) was analyzed of the subjects' DNA extracted from peripherally drawn venous blood samples (Puregene, Gentra systems, Minneapolis, MN, USA). The *SLC6A4* promoter region containing the long (16A)/short (14A) (L/S) polymorphism was PCR-amplified using the following primers: forward 5′-CGC TCC TGC ATC CCC CAT TA-3′ and reverse 5′-GGG ATG CGG GGG AAT ACT GGT-3′, which produced 297/253 bp (L/S) product. The genotype was analyzed by 3% MetaPhor (R) agarose (FMC BioProducts, Rockland, ME, USA) gel electrophoresis. Genotypic testing was conducted blindly to clinical and neuropsychological results. Since the SS and SL seem to have similar functional consequences on 5-HTT activity [8], SS and SL genotypes were combined into one group (S variant) and compared with the LL genotype (L variant).

### 2.3. Measures

#### 2.3.1. Self-Ratings

All participants were administered printed versions of the Beck Depression Inventory-II [19], the Hopelessness Scale [20], and the Beck Anxiety Inventory [21] as a self-report instrument to determine the presence and severity of depression, hopelessness, and anxiety symptoms.

#### 2.3.2. Neuropsychological Tests

An approximately 2-hour battery of 16 neuropsychological tests with standard instructions [22] was administered to all participants at 8 or 10 o'clock in the morning. The neuropsychological examination was conducted blindly to genotyping results and covered five specific cognitive domains. *Verbal reasoning* was evaluated with the similarities and *nonverbal reasoning* with the block design subtests of the Wechsler Adult Intelligence Scale-Revised (WAIS-R) [23]. Immediate and delayed *episodic memory* was assessed by the Logical Memory I and II, Verbal Paired Associates I and II, and Visual Reproduction I and II subtests of the Wechsler Memory Scale-Revised (WMS-R) [24]. *Working memory* was measured by the Visual Memory Span (forwards and backwards) and the Letter-Number Sequencing (WMS-III) [25]. The domain of *attention and executive functioning *was assessed with the Trail Making Test Part B [26], the color-word interference part of the Stroop test, and with verbal fluency both in semantic (animals) and phonological category (words beginning with the letter S) in 60 seconds. *Processing and motor speed *was assessed with the Trail Making Test Part A, the color-naming task in the Stroop test, and the Digit Symbol subtest of the WAIS-R. In addition, the simple motor speed of the right and left thumbs in 10 seconds was assessed using the Finger Tapping Test (FTT). While patients with depression are subject to multiple neuropsychological deficits, our attempt was to catch this heterogeneity by also calculating the number of impaired performances in the cognitive tests to indicate overall impairment. The impairment was defined as the number of neuropsychological test variables with at least −1 standard deviation (SD) compared with the controls' mean performance.

### 2.4. Statistical Analysis

The differences between demographic, clinical, and neuropsychological characteristics of the study groups were examined with the *χ* ^2^ test, the independent samples *t*-test, and MANCOVA age and gender as covariates. Due to a small sample size and violation of normality in sample distribution, the effect of the 5-HTTLPR variant on the neurocognitive performance was analyzed using permutational (nonparametric) MANCOVA [27, 28] gender and age as covariates. Results of permutational MANCOVA were further examined using individual permutational ANCOVA as proposed by Manly [29]. All permutational analysis was analyzed using R version 12.1 statistical environment [30] and especially permutational MANCOVA using R function Adonis in the vegan package [31]. Poisson regression [32] with the Wald chi-square statistic was examined to compare the number of impaired neuropsychological test performances related to 5-HTTLPR variants and MDD and control group interaction.

## 3. Results

Our MDD group consisted of participants with the history of recurrent depression, with the mean duration of illness being eight years. The distribution of the 5-HTTLPR variants did not differ between the patient and control groups (Table 1). Among the MDD group, 63.2% of the patients were carrying the S variant (SL or SS genotype) and 36.8% the L variant, and among controls the frequencies of S and L alleles were 68.4% and 31.6%, respectively. As shown in Table 1, the groups did not differ in terms of age, gender, or education. Overall, the MANCOVA showed significant group effect for the neuropsychological measures when age and gender were covariates (Wilks *λ* = 0.24, *F*_(19,27)_, *P* = 0.02, *η*_partial_^2^ = 0.07). Univariate comparisons revealed that MDD group scored lower than controls on the domains which represent nonverbal (visuospatial) reasoning, verbal and visual episodic memory and on the tests measuring processing and motor speed. No differences were found on the domains of working memory and executive functions.

To find out the association of the allele of the 5-HTT promoter polymorphism in depression and neurocognitive performance, we compared the interaction of the group (MDD and controls) and 5-HTTLPR variants and the neuropsychological test performances. The permutational MANCOVA revealed significant group X variant interaction in neuropsychological test results, when gender and age were covariates (*F*_(1,19)_ = 3.28, *P* = 0.01, *η*_partial_^2^ = 0.17). There was also significant group main effect (*F*_(1,19)_ = 2.49, *P* = 0.04, *η*_partial_^2^ = 0.13).

Permutational univariate comparisons (ANCOVAS) revealed that in the MDD group, the subjects with S allele scored lower in the tests of the block design (*P* = 0.04) and in the Logical Memory II (the *P* value for the interaction = 0.02). Almost significant result was reached in the Logical Memory I (*P* = 0.07) and in the semantic fluency (*P* = 0.08).

**Figure 1:**
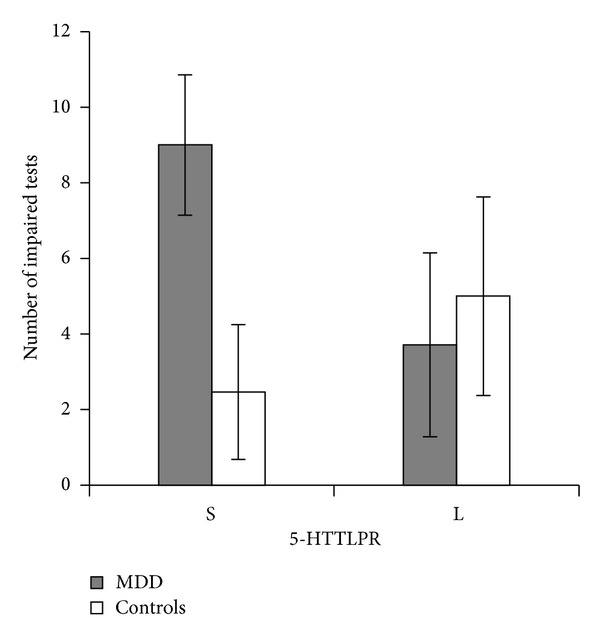
Number of impaired neuropsychological test performances in major depressive disorder (MDD) patients and controls carrying the S variant and the L variant of the 5-HTTLPR.

We also conducted Poisson regression analysis, controlling for gender and age, to compare the number of impaired neuropsychological test performances related to 5-HTTLPR variants, and MDD and control group interaction. It was found out that the mean number of impaired test performances was almost significantly (*χ* ^2^(1) = 3.04,  *P* = .081) higher in S allele carriers in the MDD group compared to controls, whereas there was no difference in L carriers between groups (Figure 1). There was also significant group main effect (*χ* ^2^(1) = 15.43, *P* < 0.001).

In the MDD group, the patients carrying the S variant did not differ from those carrying the L variant of the 5-HTTLPR in terms of the duration of the illness, number of depressive episodes, HDRS, BDI, BHS, or BAI. No significant Spearman rho correlations were found between the number of impaired neuropsychological test performances and the duration of the illness, the number of depressive episodes, HDRS, BDI, BHS, or BAI.

## 4. Discussion

Along with affective disturbances, cognitive impairment is usually one of the key dimensions in major depression. Our study was composed to find out whether depression-related neurocognitive impairment is associated with the variation in the serotonin transporter gene. As expected, the MDD group scored lower than the healthy controls on several cognitive domains representing visuospatial reasoning, episodic memory as well as processing and motor speed. Most importantly, we found out that cognitive impairment in verbal episodic memory as well as in visuospatial reasoning was associated with the short variant of the 5-HTT promoter polymorphism among patients with MDD. In addition, there was a trend showing that the overall number of impaired test performances was higher in S allele carriers in the MDD group compared with controls, whereas there was no such difference in L carriers between groups. It was notable that cognitive impairment was not related to the severity of psychiatric symptoms among the depressed patients. Thus, the result suggests that the 5-HTTLPR variation may play an important role in the modulation of neurocognitive performance in depression, the short allele being responsible for some part of the cognitive deficits seen in depression. To our knowledge, there are no previous studies examining the association between 5-HTT allelic variation and depression-related neurocognition in a real-world psychiatric setting. Some recent studies, however, support the hypothesis that the S allele interacts with stress to negatively impact cognitive functioning, especially in the older age [33, 34].

The main limitation of our study was the small sample size; even though we used a powerful candidate gene approach, our result can only be considered preliminary. Moreover, the original diallelic L/S analysis that was applied in our study can be criticized because the L allele can be subtyped into L(A) and L(G) alleles [35]. However, recent evidence comparing both diallelic and triallelic approaches has not found significant differences between the two subtype analyses in MDD patients [36]. In future studies, a haplotype analysis might be a tool for increasing the effectiveness of evaluation of the association we found [37].

The additional limitations of our study are mostly derived from the naturalistic study design. First, the patients were on several medications. Although we acknowledge that sedative psychoactive drugs may modulate neuropsychological test performances, the potential cognitive effects of these drugs could not be reliably analyzed because of the small sample size and various combinations of medication. Second, we had too few male patients to be able to analyze both genders separately, and thus our result cannot be generalized to both sexes without future studies of male patients. Genetic factors may play a greater role in the etiology of MDD in women than in men [38]. On the other hand, association between the short allele and greater reactivity to negative emotional stimuli has been shown to be independent of gender [39]. Third, information on possible early life stressors of our participants was not available.

The strength of our study is an extensive psychiatric evaluation and detailed neuropsychological test battery, performed to all participants. In our study, the cognitive measures were selected to represent the neuropsychological domains that have been shown to be the most vulnerable to impairment in depression [1]. This may partly explain the fact that we were able to reach a significant association between neurocognitive impairment and the short allele of the 5-HTT promoter polymorphism even with such a small sample. In general, a test-taking strategy and response monitoring can influence performance and obscure a genotype association in a way that may not directly reflect the underlying neurocognitive deficit [40].

While depression is not always accompanied by cognitive impairment, it is important to try to establish the factors affecting impairment to find better strategies for treatment. Our results support the suggestion according to which the impact of the 5-HTT gene on behavior might be much broader than commonly appreciated [11]. Further research is needed to confirm our findings and to indicate which neurocognitive subcomponents might be responsible for mediating neurobiological processes that are influenced by the genetic variation of the serotonin transporter. In future studies, it would also be worthwhile to further test cognition impairment related issues, 5-HTT and depression in various settings, for instance, a hypothesis according to which a risk to develop Alzheimer's disease may be increased among MDD patients with the short variant of 5-HTT, recurrent depressive episodes and prodromal neurocognitive impairment.

## 5. Conclusion

The variation in the serotonin transporter gene may play an important role in the modulation of cognitive performance in major depression, the MDD patients with the short allele of the 5-HTTLPR polymorphism being more vulnerable to cognitive impairment compared with MDD patients without the short allele inheritance.

## Figures and Tables

**Table 1:** Distribution of the 5-HTTLPR variants; demographic, clinical, and neuropsychological data for major depressive disorder (MDD) and control groups.

	MDD (*n* = 19)	Controls (*n* = 19)	Statistics	*P* value	Effect size (Cohen's *d*)
5-HTTLPR S/L allele	12/7	13/6	*χ* ^2^ = 0.12	0.732	
Gender, female/male	15/4	14/5	*χ* ^2^ = 0.15	0.703	
Age, years (range 18–56)	34.8 (12.7)	36.0 (15.8)	*t* = −2.60	0.797	0.08
Education, years	13.4 (2.4)	14.7 (2.8)	*t* = −1.47	0.150	0.50
Duration of illness, years (range)	8.1 (8.0)	—			
Number of depressive episodes	2.3 (1.6)	—			

Self-ratings:					
HDRS	24.0 (3.7)	—			
BDI total score, range 0–60	32.6 (11.8)	1.2 (1.6)	*t* = 11.46	<0.001	4.69
BHS total score, range 0–63	12.5 (5.0)	1.9 (0.9)	*t* = 9.03	<0.001	3.59
BAI total score, range 0–20	19.5 (10.6)	1.9 (1.2)	*t* = 11.48	<0.001	2.98

Neuropsychological tests:					
Abstract reasoning					
Similarities	26.8 (3.7)	27.3 (3.0)	*F* = 0.15	0.700	0.15
Block design	34.5 (7.6)	39.5 (8.6)	*F* = 5.19	0.029	0.62
Episodic memory					
Logical memory I	22.3 (8.6)	27.4 (5.4)	*F* = 4.69	0.037	0.73
Logical memory II	19.7 (8.4)	25.0 (4.9)	*F* = 5.27	0.028	0.80
Verbal paired associates I	18.6 (4.7)	20.7 (2.6)	*F* = 3.06	0.089	0.58
Verbal paired associates II	6.8 (1.6)	7.4 (0.9)	*F* = 2.74	0.107	0.48
Visual reproduction I	33.3 (3.7)	38.6 (2.1)	*F* = 32.08	<0.001	1.83
Visual reproduction II	32.5 (5.1)	37.7 (2.9)	*F* = 18.01	<0.001	1.30
Working memory					
Visual memory span	18.3 (2.9)	19.6 (2.4)	*F* = 2.37	0.133	0.49
Letter number sequencing	10.5 (2.4)	11.3 (2.0)	*F* = 1.20	0.280	0.36
Executive functioning					
Trail making test B sec.	74.1 (21.6)	71.3 (26.9)	*F* = 0.15	0.697	0.12
Stroop color-word sec.	109.5 (21.3)	104.9 (30.2)	*F* = 0.30	0.587	0.18
Semantic fluency	23.2 (6.3)	26.5 (5.1)	*F* = 3.13	0.086	0.58
Letter fluency	16.8 (3.9)	18.3 (3.7)	*F* = 1.18	0.290	0.39
Processing and motor speed					
Trail making test A sec.	37.6 (10.8)	31.4 (8.0)	*F* = 5.38	0.026	3.96
Stroop color naming	73.1 (11.7)	63.5 (10.2)	*F* = 7.01	0.012	0.88
Digit symbol (WAIS-R)	57.6 (11.9)	63.5 (12.1)	*F* = 3.04	0.090	0.49
Finger tapping, dominant	47.5 (4.6)	51.1 (6.1)	*F* = 4.92	0.033	0.67
Finger tapping, nondominant	41.7 (5.1)	45.5 (4.4)	*F* = 5.71	0.022	0.80

The results are given as mean (SD), except for gender and 5-HTTLPR variants, for which the results are given as frequency. Group comparisons in neuropsychological tests were analyzed with MANCOVA in which gender and age were covariates.

HDRS: Hamilton Rating Scale for Depression; BDI: Beck's Depression Inventory; BHS: Beck's Hopelessness Scale; BAI: Beck's Anxiety Inventory.
